# Quality of Health Information on the Internet for Urolithiasis on the Google Search Engine

**DOI:** 10.1155/2016/8243095

**Published:** 2016-12-04

**Authors:** Dwayne T. S. Chang, Robert Abouassaly, Nathan Lawrentschuk

**Affiliations:** ^1^Department of Urology, Sir Charles Gairdner Hospital, Nedlands, WA, Australia; ^2^Urology Institute, University Hospitals Case Medical Center, Cleveland, OH, USA; ^3^Department of Urology, Austin Hospital, Heidelberg, VIC, Australia; ^4^Olivia Newton-John Cancer Research Institute, Austin Hospital, Heidelberg, VIC, Australia

## Abstract

*Purpose*. To compare the quality of health information on the Internet for keywords related to urolithiasis, to assess for difference in information quality across four main Western languages, and to compare the source of sponsorship in these websites.* Methods*. Health On the Net (HON) Foundation principles were utilised to determine quality information. Fifteen keywords related to urolithiasis were searched on the Google search engine. The first 150 websites were assessed against the HON principles and the source of sponsorship determined.* Results*. A total of 8986 websites were analysed. A proportion of HON-accredited websites for individual search terms range between 2.5% and 12.0%. The first 50 websites were more likely to be HON-positive compared to websites 51–100 and 101–150. French websites searched were more likely to be HON-positive whereas German websites were less likely to be HON-positive than English websites. There was no statistically significant difference between the rate of HON-positive English and Spanish websites. The three main website sponsors were from government/educational sources (40.2%), followed by commercial (29.9%) and physician/surgeon sources (18.6%).* Conclusions*. Health information on most urolithiasis websites was not validated. Nearly one-third of websites in this study have commercial sponsorship. Doctors should recognise the need for more reliable health websites for their patients.

## 1. Introduction

The Internet is a convenient source of health-related information for patients. In 2014, 87% of adults in America use the Internet and among them and 72% used it to look for health information [1, 2]. Thirty-five percent of American adults attempted to diagnose a medical condition on themselves or others by seeking health information on the Internet [1]. It is thus crucial to assess the validity of health information available on the Internet and improve on any deficiencies. The quality of health information on the Internet was found to be variable in the fields of oncology, urological oncology, and benign prostatic hyperplasia [3–5]. There is also a growing presence of urologists in the Internet and online social media which may overwhelm a lay person with the breadth of information or even cause confusion and misunderstanding with conflicting views particularly on controversial topics [6]. Furthermore, websites with a sole focus on complications arising from surgical procedures are not uncommon [7] and this may provide biased information.

Urolithiasis is an increasingly common disease affecting approximately 1 in 11 American adults (8.8%) [8]. There are medical and surgical treatments for this disease; the decision for a mode of therapy should be made in consultation with a urologist. Patients who choose to self-manage their disease based on health information on the Internet may be confused by the vast amount of unregulated information which may also be biased.

There are services available to recognise valid and reliable online health information. Health On the Net (HON) Foundation is a nonprofit, nongovernmental organisation accredited by the Economic and Social Council of the United Nations to certify suitable websites that provide quality, objective, and transparent medical information. It is currently the most widely accepted certification tool used by publishers of health information websites [9].

Our objectives were (1) to compare the quality of health information on the Internet for keywords related to urolithiasis, (2) to assess for difference in information quality across four main Western languages (English, French, German, and Spanish), and (3) to compare the source of sponsorship in these websites.

## 2. Methods

Our methodology was previously described and used [3–5]. We used the Google search engine (https://www.google.com/) to search for 15 keywords related to urolithiasis. These keywords were “Renal colic”; “Kidney stone”; “Ureteric stone”; “Bladder stone”; “Staghorn calculus”; “Ureteral stent”; “Lasertripsy”; “Extracorporeal shock wave lithotripsy”; “Percutaneous nephrolithotomy”; “Retrograde intrarenal surgery”; “Litholapaxy”; “Medical expulsive therapy”; “Calcium calculus”; “Uric acid calculus”; and “Cystine calculus” and the respective equivalent terms in French, German, and Spanish. These terms were translated from English using professional medical translation services and the accuracy confirmed with doctors who have French, German, or Spanish as their primary language. “Sponsored links” presented by the Google search engine either at the top of the search page or on the side under a banner were not included throughout this entire study.

To reduce the risk of missing out on relevant websites, the first 150 websites found from each search were each screened for quality of information based on HON principles (Table 1). The Foundation's HONcode web browser toolbar (available from http://www.hon.ch/) was used on a personal computer. An indicator on the toolbar lights up automatically if the website on view is accredited by the HON Foundation. The HONcode toolbar was used in several studies and appears to be a valid and reliable tool [3–5].

An analysis of website sponsorship was carried out for all keywords in English. Websites were determined to be sponsored by (1) lawyers, (2) nonprofit organisations, (3) government/educational institutions, (4) commercial, (5) surgeons/physicians, (6) other health professionals, or (7) other sources. If the source of sponsorship was not obviously apparent, the website was explored by two examiners (DC and NL) until a definite source could be determined.

For quality control, nonaccredited websites found in an English search for “Renal colic” were manually evaluated to determine if they adhere to the HON Foundation principles (Table 1), despite not being “officially” certified by the HON Foundation [9].

The first 150 websites found for each term were divided into tertiles (first, middle, and last 50 search results). The proportion of HON-accredited websites within each tertiles was analysed and compared using the chi-squared test. This analysis determines whether HON-accredited websites were more likely to appear in the first, middle, or last tertile of search results. The proportions of accredited websites were compared across search terms and languages using the chi-squared test (or Fisher exact tests when cell counts were less than five). All statistical tests were two-sided and statistical significance was defined as *P* < 0.05. Logistic regression analysis was performed using the variables of search term, language, and tertiles of search results. The referent groups for each variable were the English keywords and the first 50 websites as these have the highest proportion of HON-accredited websites. Odds ratios and 95% confidence intervals were calculated from the logistic regression analysis. Analyses were performed using SAS 9.3 (SAS Institute Inc., Cary, NC, USA).

## 3. Results

**Figure 1:**
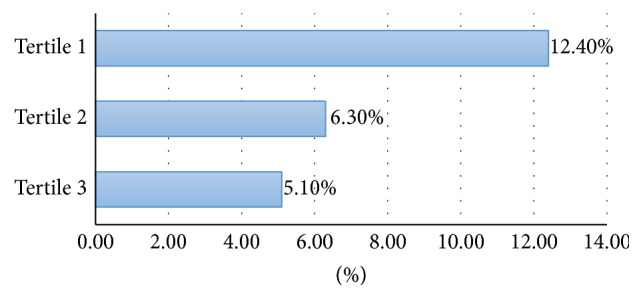
Rates of HON-accredited websites within each tertile group.

**Figure 2:**
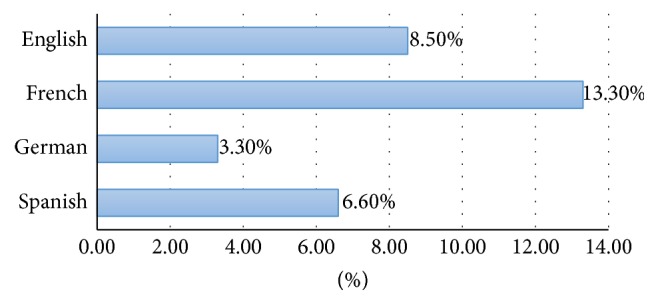
Total rates of HON-accredited websites among the main Western languages.

Only 136 websites were found for the German search term for “medical expulsive therapy” instead of 150 thus a total of 8986 websites were analysed instead of the projected 9000 websites. In total, 712 (7.9%) of websites were HON-accredited. Eleven out of 15 search terms had less than 10% of HON-accredited websites (Table 2). A proportion of HON-accredited websites for individual search terms range between 2.5% (litholapaxy) and 12.0% (renal colic). The first 50 websites searched were more likely to be HON-positive compared to websites 51–100 (12.4% versus 6.3%; *P* < 0.01) and 101–150 (12.4% versus 5.1%; *P* < 0.01) (Figure 1). In comparison with English websites, French websites searched were more likely to be HON-positive (13.3% versus 8.5%; *P* < 0.01) whereas German websites were less likely to be HON-positive (3.3% versus 8.5%; *P* < 0.01). There was no statistically significant difference between rate of HON-positive English and Spanish websites (8.5% versus 6.6%; *P* = 0.26) (Figure 2). The three main website sponsors were from government/educational sources (40.2%), followed by commercial (29.9%) and physician/surgeon sources (18.6%). Among the different sources of sponsorship, websites sponsored by not-for-profit sources had the highest rate of HON accreditation (27.3%), followed by those sponsored by commercial (11.0%), government/education (8.8%), and physician/surgeon sources (4.5%) (Table 3). The number of HON-accredited websites (for the English search for “renal colic”) found by manually applying the HON Foundation principles correlated with the number found via using the HONcode web browser toolbar.

## 4. Discussion

In total, less than one in twelve websites in this study were accredited by the HON Foundation. Similar findings were previously demonstrated in a recent study relating to benign prostatic hyperplasia [5]. Higher rates of HON accreditation in the region of 20% were reported in previous oncological studies [3, 4]. These collective findings elicit concerns on the significant likelihood of patients encountering invalid or biased medical information when searching the Internet for information on their diseases. Furthermore, there is significant variability in the rate of HON-accredited websites even among closely related search terms for the same condition (Table 2). This issue has been recognised by two of the foremost urological associations in the world; the American Urological Association (AUA) and the European Association of Urology (EAU). The AUA has a website containing patient information resources written and reviewed by urologists [10]. Similarly, the EAU has a website providing unbiased and comprehensive information for patients on common conditions ranging from kidney, bladder, and prostate cancers to urolithiasis and lower urinary tract symptoms [11].

In terms of website sponsorship, not-for-profit websites had the highest rate of HON accreditation, followed websites sponsored by commercial, government/education, and physician/surgeon sources (Table 3). It is sobering to find that websites sponsored by commercial sources had a higher rate of HON accreditation than websites sponsored by physician/surgeon sources. This illustrates the potentially strong influence of commercial bias on the distribution of medical information to the general population. A possible explanation of the relatively lower HON-accreditation rate with physician/surgeon sponsorship is that a proportion of these websites have content with the purpose of “promoting” their services instead of providing unbiased clinical information for patients. Another possibility is that commercial sponsors are more likely to be able to bear the financial costs associated with maintaining accreditation of sponsored websites. However, it is important to note that “for-profit” websites pay a higher fee than “not-for-profit” websites (between EUR160 and EUR325 versus EUR50 and EUR160, depending on popularity of the website) [12].

HON is not the only method of validating the reliability and quality of health information on websites. In 2000, an e-Health Code of Ethics was developed in a summit hosted by the World Health Organisation and the Pan-American Health Organisation [13]. This code of ethics has been translated into five other languages as well (http://www.ihealthcoalition.org/ehealth-code-of-ethics/). Another useful tool is the DISCERN instrument (http://www.discern.org.uk/) developed with support from The British Library and the National Health Service Executive Research & Development Programme [14]. Although these other instruments were likely to be useful, the HON Foundation principles were used in this study instead due to the ease of use. It was relatively convenient to have an automated toolbar feature which allowed rapid and accurate processing of websites, especially when working with thousands of websites such as in this study. We could not find similar features with other publically available validation tools to allow us such convenience. It is also important to note that although HON is not the only method of website validation, if a website is validated to conform to the principles of the HON Foundation (Table 1), that website can be assured to provide quality and unbiased health information.

This study has several limitations. Firstly, HON accreditation may not be sought universally by all health-related websites possibly due to lack of knowledge of its existence or benefits. Another possible explanation is the cost associated with maintaining a HON-accredited website. The initial request for HON certification is free of charge and is valid for one year. Renewal and extension of the HON certification involve paying a membership fee depending on the type and popularity of the website. Certification is free of charge in the first year and costs between EUR50 and EUR325 per year subsequently to maintain the certification, depending on the type and popularity of websites [12]. These factors may increase the risk of false negatives as not all websites lacking HON accreditation provide invalid or biased information. It is important to highlight that payment of the renewal fee does not guarantee renewal of the HON certification [12]. If the HON Foundation review team finds that the website has become noncompliant to their principles, the payment is refunded if it has already been paid [12]. This minimises the conflict of interest for the recertification process. The team reassesses a website every year that there is a request to renew the HON certification, so there is no blind approval of a website without proper review.

In addition, the Internet is dynamic; thus the listing of websites according to popularity on the search engine may change from day to day. In effect, it is possible for movement of websites across tertile groups; for example, a website ranked 51 today may be ranked 49 the next day. Although we used the Google search engine because it is the most popular and widely used, there are other search engines available and each of these may rank websites differently according to their algorithms; thus a similar study on a different search engine may yield different results due to movement of websites across tertile groups. In this study, all website cookies and data cache were deleted prior to searching for websites to minimise the impact of browsing history on future searches. However, patients may not routinely take this precaution; thus their previous use may potentially influence their search results. Furthermore, it is important to note that the HONcode tool used does not reflect the scientific quality of website content, but rather the reliability of the website and its authors.

## 5. Conclusions

In this study, only a minority of websites found relating to urolithiasis were validated by the HON Foundation. There were discrepancies in accreditation rate across various search terms and major Western languages. There was significant potential commercial bias found among the websites in this study as a sizeable proportion had commercial sponsorship. In this study, rates of HON accreditation of websites sponsored by doctors were no better than those sponsored commercially, possibly due to the cost of maintaining HON certification. Our patients need guidance to navigate the ocean of information on the Internet and we should help direct them to websites known to provide reliable and unbiased information.

## Figures and Tables

**Table 1:** Principles of the HON Foundation.

Principles	Criteria (summary)
Authoritative	Indicate the qualifications of the authors
Complementarity	Information should support, not replace, the doctor-patient relationship
Privacy	Respect the privacy and confidentiality of personal data submitted to the site by the visitor
Attribution	Cite the source(s) of published information, date medical and health pages
Justifiability	Site must back up claims relating to benefits and performance
Transparency	Accessible presentation, accurate email contact
Financial disclosure	Identify funding sources
Advertising policy	Clearly distinguish advertising from editorial content

Note: reproduced with permission from the Health On the Net Foundation.

**Table 2:** Rates of HON-accredited websites among the search terms.

Search term	Total websites	HON+	HON−	%HON+
Renal colic	652600	72	528	12.0
Kidney stone	4977000	61	539	10.2
Ureteric stone	318580	62	538	10.3
Bladder stone	9518730	45	555	7.5
Staghorn calculus	65820	41	559	6.8
Ureteral stent	670497	42	558	7.0
Lasertripsy	19127	34	566	5.7
Extracorporeal shock wave lithotripsy	327270	60	540	10.0
Percutaneous nephrolithotomy	191010	45	555	7.5
Retrograde intrarenal surgery	142220	45	555	7.5
Litholapaxy	83850	15	585	2.5
Medical expulsive therapy	585436	33^*∗*^	553^*∗*^	5.6
Calcium calculus	1414373	51	549	8.5
Uric acid calculus	354410	52	548	8.7
Cystine calculus	145308	54	546	9.0

Note: HON+, HON-accredited; HON−, non-HON-accredited; %HON+, percentage of HON-accredited websites out of a total of 600 websites (150 per search term × 4 languages).

^*∗*^Only 136 websites found for the German search term, thus totalling 586 websites for four languages instead of 600.

**Table 3:** Rates of sponsorship and HON-accredited websites within each sponsor.

Sponsorship	HON+	HON−	%HON+
Lawyer	0	0	∞
Not-for-profit	18	48	27.3
Government/education	80	825	8.8
Commercial	74	598	11.0
Other health professionals	0	69	0.0
Physician/surgeon	19	399	4.5
Others	0	120	0.0

Note: HON+, total of HON-accredited websites; HON−, total non-HON-accredited websites; %HON+, percentage of HON-accredited websites according to each sponsorship source.
